# Characterization of Cyclin E Expression in Multiple Myeloma and Its Functional Role in Seliciclib-Induced Apoptotic Cell Death

**DOI:** 10.1371/journal.pone.0033856

**Published:** 2012-04-25

**Authors:** Liat Josefsberg Ben-Yehoshua, Katia Beider, Avichai Shimoni, Olga Ostrovsky, Michal Samookh, Amnon Peled, Arnon Nagler

**Affiliations:** Department of Hematology and Bone Marrow Transplantation, Chaim Sheba Medical Center, The Guy Weinshtock Multiple Myeloma Foundation, Tel-Hashomer, Israel; Virginia Commonwealth University, United States of America

## Abstract

Multiple Myeloma (MM) is a lymphatic neoplasm characterized by clonal proliferation of malignant plasma cell that eventually develops resistance to chemotherapy. Drug resistance, differentiation block and increased survival of the MM tumor cells result from high genomic instability. Chromosomal translocations, the most common genomic alterations in MM, lead to dysregulation of cyclin D, a regulatory protein that governs the activation of key cell cycle regulator – cyclin dependent kinase (CDK). Genomic instability was reported to be affected by over expression of another CDK regulator - cyclin E (CCNE). This occurs early in tumorigenesis in various lymphatic malignancies including CLL, NHL and HL. We therefore sought to investigate the role of cyclin E in MM. CCNE1 expression was found to be heterogeneous in various MM cell lines (hMMCLs). Incubation of hMMCLs with seliciclib, a selective CDK-inhibitor, results in apoptosis which is accompanied by down regulation of MCL1 and p27. Ectopic over expression of CCNE1 resulted in reduced sensitivity of the MM tumor cells in comparison to the paternal cell line, whereas CCNE1 silencing with siRNA increased the cell sensitivity to seliciclib. Adhesion to FN of hMMCLs was prevented by seliciclib, eliminating adhesion–mediated drug resistance of MM cells. Combination of seliciclib with flavopiridol effectively reduced CCNE1 and CCND1 protein levels, increased subG1 apoptotic fraction and promoted MM cell death in BMSCs co-culture conditions, therefore over-coming stroma-mediated protection. We suggest that seliciclib may be considered as essential component of modern anti MM drug combination therapy.

## Introduction

Multiple myeloma (MM) is a malignancy of antibody-secreting B cells. Upon transformation plasma cells accumulate within the bone marrow (BM). The symptoms of this disease relate to anemia, immunosuppression, bone destruction, and renal failure [1], [2], [3]. The disease is initially chemo-sensitive and may enter dormant phases of variable duration. With time, it develops independence from microenvironment-growth factors and resistance to medication and apoptosis [4]. At this stage, malignant cells may be propagated in vitro to produce human myeloma cell lines (hMMCLs). Elucidation of the genetic events related to the various stages of development is crucial to understanding the pathogenesis of this disease.

Genetic profiling of MM tumor cells reveals karyotypic instability which is characterized by aneuploidy including hyperploidy, hypoploidy, trisomy or tetrasomy, monosomy or nullosom as well as other chromosomal abnormalities such as translocations, deletions, and amplifications [5]–[7]. High prevalence (37%) of near tetraploid plasma cells was detected in BM samples from MM patients [7]. This instability is detected at the earliest stages of the disease and increases to extreme genetic abnormalities, similar to those observed for solid tumors [8].

The frequency and the extent of karyotypic abnormalities correlate with the development of the disease and the response to treatment. Abnormal karyotype is found in about 20% of patients at stage I, 60% of patients at stage III, and more than 80% of patients with an extramedullary tumor [6]. The karyotipic anomalies in hMMCLs are greater than 90% [6].

Additionally, chromosome 13 abnormalities have been reported to occur in MM and to have an unfavorable prognosis in MM [5], [9]–[11].

About 40% of MM tumors can be classified by commonly observed mutations that include chromosomal translocations involving the immunoglobulin gene (Ig) with 5 recurrent chromosomal partners and oncogenes: 11q13 (CCND1); 4p16 (FGFR3 and MMSET); 6p21 (CCND3); 16q23 (MAF); and 20q11 (MAFB) [8]. Recently, a strong association between Δ13 and translocation t(11;14) with deletions of the IgH was reported [12]. The high genetic heterogeneity which affects the disease initiation, progression, and therapeutic response presents a major challenge in the treatment of MM.

Genomic instability was reported to be affected by over-expression of cyclin E (CCNE). Cyclin E is the regulatory subunit of Cyclin Dependent Kinase 2 (Cdk2), a heterodimer that functions as a key regulator of cell cycle progression. This heterodimer governs the G1-S boundary, where initiation of DNA replication, S-phase specific transcription and centrosome duplication occur. Periodic expression at the G1-S boundary of cyclin E is regulated by its transcription followed by rapid turnover [13], [14]. Deregulation results in untimely expression of CCNE which may induce genomic instability [15]. Indeed, its over expression is detected as an early event in tumorgenesis of many solid tumors, including breast, colon, and prostate carcinomas, as well as hematologic malignancies like chronic lymphocytic leukemia and Hodgkin’s and non-Hodgkin’s lymphoma [14], [16], [17]. As CCND has been found to be overexpressed in more than 80% of MM tumors [18], it may play a critical role in the development, proliferation and survival of MM cells. Thus MM cells can serve as good candidates for the examination of CDK inhibition.

The development of CDK inhibitors for chemotherapeutic use has been a favorable target of the pharmacological industry [19]. Whereas, the first generation of such inhibitors had a broad range of CDK targets, current development aims for more specific inhibition and higher affinity compounds. Several such inhibitors have been tested in-vitro in tumor cell lines and in-vivo in animal models of various neoplasms [20]. MM has been found to be sensitive to a range of CDK inhibitors [21]–[25]. Seliciclib (Roscovitine, CYC202) a selective inhibitor of CDK’s has potent cytotoxicity against MM cells [25], [26]. The suggested mechanism of action is modulation of MCL1 (an antiapoptotic protein in myeloid cell leukemia) mediated by inhibition of IL-6 secretion in the BM milieu [25], [26]. Therefore, this inhibitor dually targets the tumor cells as well as the microenvironment that supports its growth. Currently there are several ongoing clinical trials utilizing an oral formulation of seliciclib, the initial Phase I study reported a stabilization of various solid tumors accompanied with grade 2–3 toxicities [27]. Seliciclib has been reported to inhibit CCND expression [20], [28] and therefore may have an additional advantage in the treatment of MM that over express this protein [18].

In this work we set out to determine the role of cyclin E in the proliferation and survival of MM cells. We chose to conduct the study on seven different hMMCL’s in order to signify the heterogeneity of this disease. Our goals were: to determine the expression profile of cyclin E; to assess the sensitivity of hMMCLs to the CDK inhibitor seliciclib; to elucidate the mechanism of seliciclib-induced-apoptosis and to explore the CDK-inhibition on the adhesion of MM cell to an extracellular matrix (ECM).

## Results

### The Expression Profile of CCNE1 in Human MM Cell Lines (hMMCLs)

**Figure 1:**
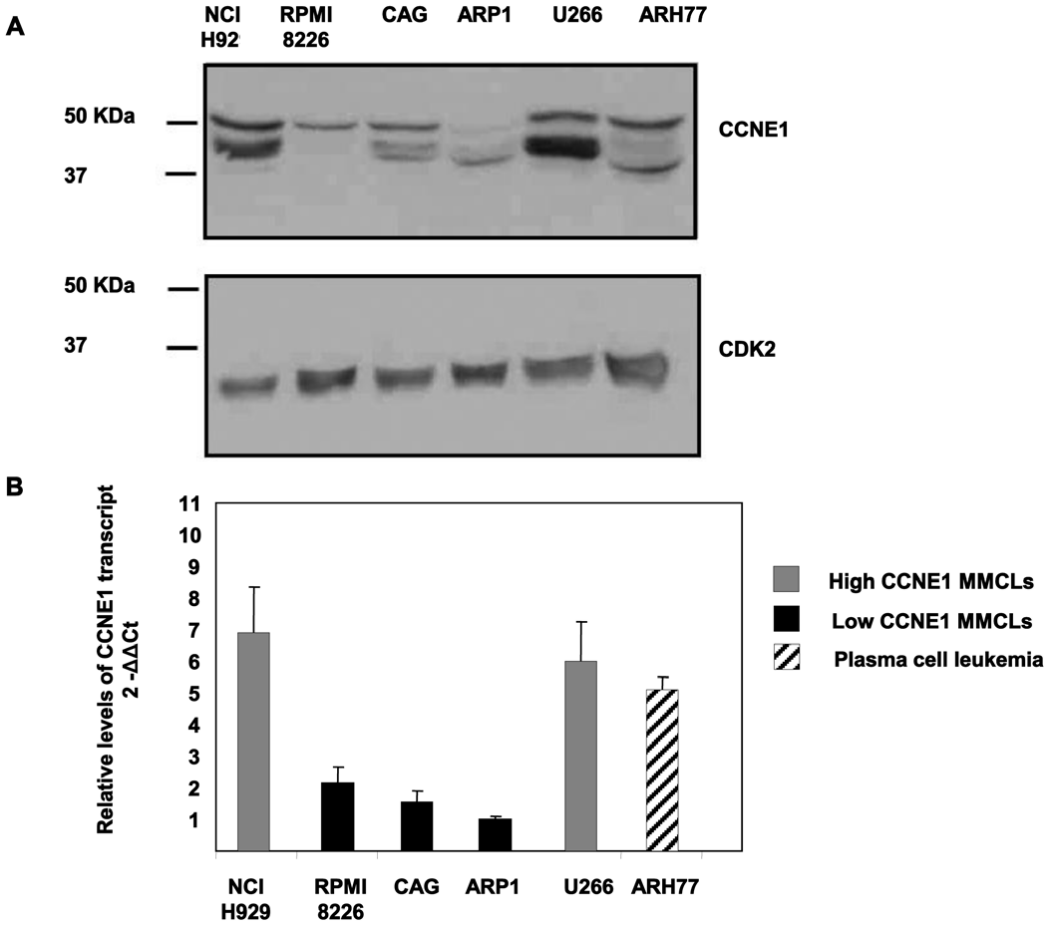
CCNE1 expression profile in hMMCLS. (A) Various multiple myeloma cell lines (NCI H929, RPMI8226, CAG, ARP1 and U266) and plasma cell leukemia cell line ARH77 in logarithmic growth phase were extracted and subjected to immunoblotting, utilizing CCNE1 antibodies. CDK2 expression served as an internal loading control. (B) Various multiple myeloma cell lines (NCI H929, RPMI8226, CAG, ARP1 and U266) and plasma cell leukemia cell line ARH77 in logarithmic growth phase were extracted and subjected to qRT-PCR in quadruplicates. Data are represented as mean±standard deviation of the ratio of CCNE1 and GAPDH between all hMMCLs and ARP1. (A–B) Experiments were performed at least 3 times and one representative result is presented.

Multiple myeloma displays a high genomic instability [8] which is observed in other types of cancer and is known to be affected by over-expression of cyclin E [14], [16]. To determine the expression profile of CCNE1 in hMMCLs, cells were extracted, and the level of the protein verified by immunoblotting (Figure 1A). The levels of CDK2 expression served as an internal control. CCNE1 was detected mainly as a 50 kDa protein. Additional lower molecular weight bands of 40 kDa, were observed in agreement with previous studies [29].

Further analysis of the CCNE1 transcript level was carried out by quantitative reverse transcription PCR (qRT-PCR) of RNA that was extracted from free running hMMCLs. The levels of CCNE1 transcript were compared to the level of GAPDH that served as an internal control. The levels of protein and the transcript were directly proportional (Figure 1B). We thus defined the hMMCLs according to the level of CCNE1 expression: high (U266 and NCI-H929) and low (CAG, RPMI8226 and ARP-1). The plasma cell leukemia cell line ARH77 displayed high level of CCNE1.

### The Dependence of the Multiple Myeloma Cells Survival on the CDK Activity

**Figure 2:**
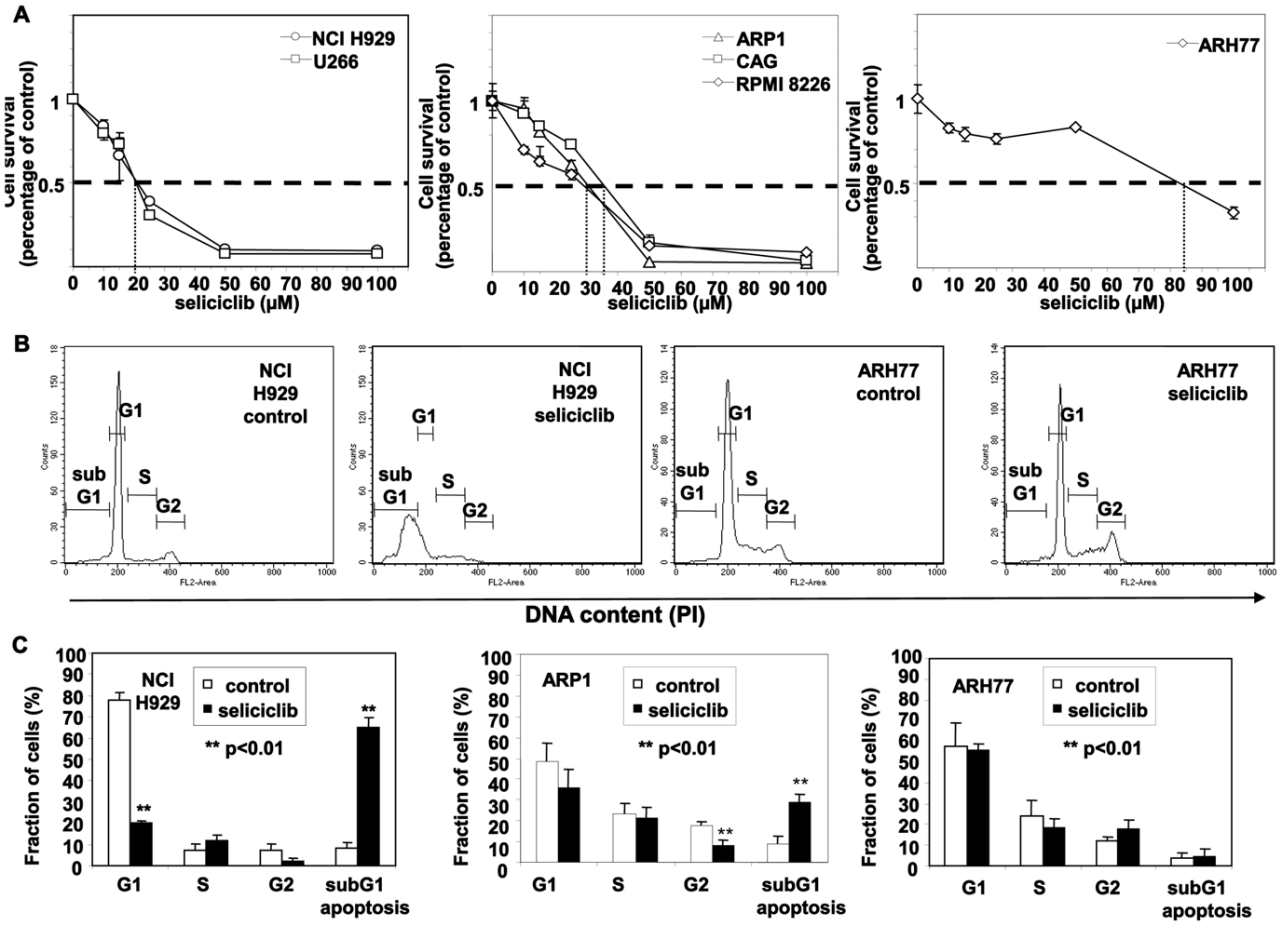
Heterogenous resistancy to seliciclib in hMMCLs. (A) The indicated hMMCLs were incubated in the absence or presence of increasing concentrations of seliciclib for 3 days. Cell viability was determined by MTT assay. Data are represented as mean±standard deviation. Experiments were performed at least 3 times and one representative result is presented. Seliciclib resulted in a decrease in cell viability with an IC50 ranging from 25 to 90 µM. (B) Cell cycle analysis by PI staining was performed on hMMCLs incubated in the absence or presence of 50 µM seliciclib for 12 hours. Cells were collected, fixed, stained with propidium iodide (PI) and analyzed by flow cytometry. DNA distribution in the cells is presented. (C) Quantification of cell cycle stage analysis of control and seliciclib-treated (50 µM, 12 hours culture) hMMCLs. Analysis of representative lines of highly sensitive (NCI H929), a moderately-sensitive (ARP1) and a resistant (ARH77) lines are displayed. The subdyploid DNA peak (subG1) represents apoptotic cell fraction. Data are represented as mean±standard deviation of 3 different experiments. Probability values of t-test are presented **p<0.01.

Previous studies have reported that hMMCLs are sensitive to inhibitors of CDK. Seliciclib (Roscovitine, CYC202), is a selective chemical inhibitor with potent cytotoxicity against MM cells [25], . To determine its effect, six MM cell lines (NCI H929, RPMI8226, CAG, ARP-1 and U266) and one plasma cell leukemia cell line ARH77 were incubated in the presence of either DMSO or increasing concentrations of seliciclib for 72 hours. The viability of the cells was determined by an MTT assay. There was no difference in MTT incorporation between control-untreated cells and DMSO-treated cells (data not shown). Treatment with seliciclib resulted in dose-dependent cytotoxicity (Figure 2A), with a calculated IC50 ranging from 20 to over 80 µM (Table 1). As demonstrated in Figure 2A, response of hMMCLs to seliciclib correlated to CCNE1 expression levels. High CCNE1-expressing NCI H929 and U266 cells revealed high sensitivity to seliciclib, whereas low-CCNE1 cells (ARP-1, CAG and RPMI8226) showed intermediate sensitivity. ARH77 cells were different from the other cell lines and despite the high level of CCNE1 were resistant to seliciclib.

**Table 1:** Seliclib sensitivity in MMCLs.

MMCL	IC50 (µM)
ARH77	84
ARP1	32
CAG	33
MM	38
NCI H929	24
RPMI 8226	33
U266	26

Various multiple myeloma cell lines (NCI H929, RPMI8226, CAG, ARP1 and U266) and plasma cell leukemia cell line ARH77 were incubated in the absence or presence of increasing concentrations of seliciclib for 3 days. Cell viability was determined by MTT assay. The calculated average value of IC50 of at least 3 experiments is presented.

To characterize the cytotoxic effect of seliciclib on MM cells, we performed cell-cycle analysis on representative hMMCLs from each category; similar results were obtained for all cell lines (data not shown). Analysis of nuclear DNA distribution using flow cytometry showed that in sensitive NCI H929 cells seliciclib treatment augmented the fraction of hypoploid cells (subG1 cells),an established indication of apoptosis, resulting from degradation and subsequent leakage of nuclear DNA from the cells. An increase in the subG1 cell population following seliciclib treatment was accompanied by a concomitant reduction in the G0/G1 cell population. In contrast, the cell cycle distribution of the resistant ARH77 cells was not affected by seliciclib (Figure 2B). Statistical analysis of multiple experiments with two sensitive lines (NCI H929 and ARP1) revealed a significant difference in the fraction of apoptotic cells between control and seliciclib treated cells (Figure 2C).

**Figure 3:**
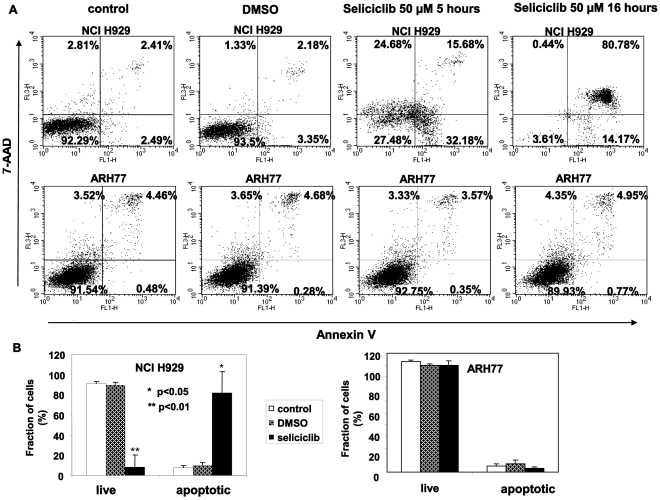
Seliciclib induces apoptosis in sensitive hMMCLs. hMMCLS were incubated in the absence or presence of 50 µM seliciclib. Following 5 hours or 16 hours of culture cells were collected, fixed, stained using annexin V/PI and analyzed by flow cytometry. The extent of apoptosis is expressed as a percentage of cells positively stained using annexin V. (A) Seliciclib-sensitive NCI H929 and seliciclib-resistant ARH77 were incubated in the presence or absence of seliciclib for the indicated time points. Experiments were performed at least 3 times and one representative result is presented. (B) Representative analysis of seliciclib-induced apoptosis following a 16 hour incubation time in control, DMSO and seliciclib treated cells. The percentage of live (annexin V/PI-negative) and apoptotic (annexin V-positive) cells is given. Data represents the mean and standard deviation of 3 different experiments. Probability values of t-test are presented *p<0.05 **p<0.01.

Further characterization was carried out by quantification of annexin V binding as a marker for early apoptosis. The sensitivity of the various hMMCLs strains varied from the resistant ARH77 line, to the highly sensitive NCI H929. The results for 2 representative cell lines are depicted in figure 3. Annexin V binding was the same for control-untreated cells and DMSO-treated cells (Figure 3A). Following seliciclib treatment the sensitive line NCI H929 line showed an increased fraction of annexin V positive cells, while the resistant line ARH77 showed no annexin V binding (Figure 3A). Annexin V binding was detected within 5 hours of seliciclib addition, and it reached a maximum following overnight incubation (Figure 3A). Statistical analysis of multiple experiments revealed a statistically significant induction of apoptosis following seliciclib treatment (Figure 3B), only in the sensitive line (p<0.05).

**Figure 4:**
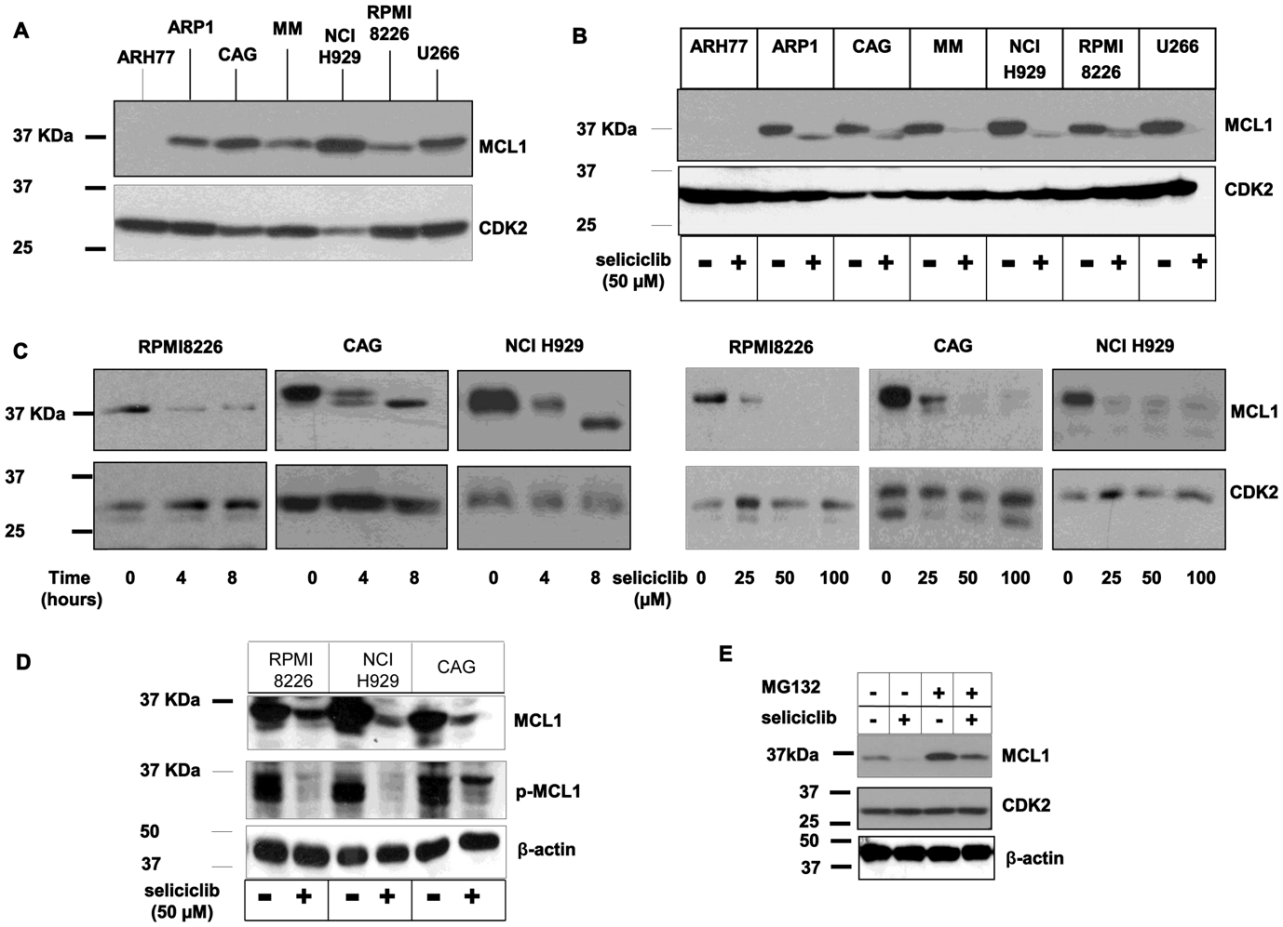
Seliciclib downregulates MCL1 expression in hMMCLs. (A) The indicated multiple myeloma cell lines in logarithmic growth phase were extracted and subjected to immunoblotting, utilizing MCL1 antibodies. In all experiments CDK2 expression serves as an internal loading control. Experiments were performed at least 3 times and one representative result is presented. (B–C) MCL1 downregulation by seliciclib. Cells were extracted and subjected to immunoblotting. (B) Various hMMCLs were incubated in the presence of seliciclib 50 µM or DMSO for 6 hours and the level of MCL1 expression was analyzed. (C) RPMI8226, CAG and NCI H929 cells were incubated in the absence or presence of seliciclib 50 µM for the indicated time points, or in the presence of increasing concentrations of seliciclib for 8 hours. (D) Reduction in MCL1 phosphorylation by seliciclib. Cells were treated with 50 µM or DMSO for 8 hours and the levels of total and phosphorylated MCL1 were analyzed by immunoblotting using specific antibodies. β-actin was used to confirm equal protein loading. (E) CAG cells were incubated in the absence or presence of seliciclib or MG132 (10 µM) exclusively or combined. MCL1 level of expression was verified by immunoblotting. β-actin was used to confirm equal protein loading.

Investigation of seliciclib-mechanism of action was pursued at the molecular level. This was achieved by analysis of the expression of MCL1, an antiapoptotic BCL2 family member, that is constitutively expressed in multiple myeloma cells [30]. To determine the expression level of MCL1 in hMMCLs, cells were extracted, and protein expression was verified by immunoblotting. Interestingly, the basal level of MCL1 expression was considerably lower in ARH77 in comparison to the other hMMCLs tested (Figure 4A). This result suggests that other antiapoptotic BCL2 family member may play a role in the survival of this particular cell line.

Incubation of the cells with seliciclib caused a remarkable reduction of MCL1 expression for all hMMCLs tested (Figure 4B). The reduction was time and dose-dependent (Figure 4C).

Careful analysis of the expression profile, following seliciclib addition, revealed lower molecular forms of MCL1 possibly reflecting hypo-phosphorylation (Figure 4C) in agreement with previous studies [31].

To evaluate the effect of seliciclib on MCL1 phosphorylation in hMMCLs we tested the levels of phosphorylated MCL1 in control and seliciclib-treated cells using phospho-Ser159/Thr163 MCL-1 specific antibody. Indeed, MCL1 phosphorylation was remarkably reduced following seliciclib treatment (Figure 4D). These results clearly indicate the role of seliciclib in the loss of total and phosphorylated MCL1 protein.

To further explore the seliciclib-induced down regulation of MCL1 we incubated the cells in the presence of both seliciclib and MG132, a potent proteasome inhibitor. Indeed, in the presence of both inhibitors, the seliciclib-induced reduction of MCL1 expression was eliminated. The level of MCL1 was comparable to control untreated cells (Figure 4E). Therefore we can conclude that seliciclib leads to downregulation of MCL1 protein via proteasome-mediated degradation.

### The Effect of Seliciclib on the Cell Cycle Regulators

**Figure 5:**
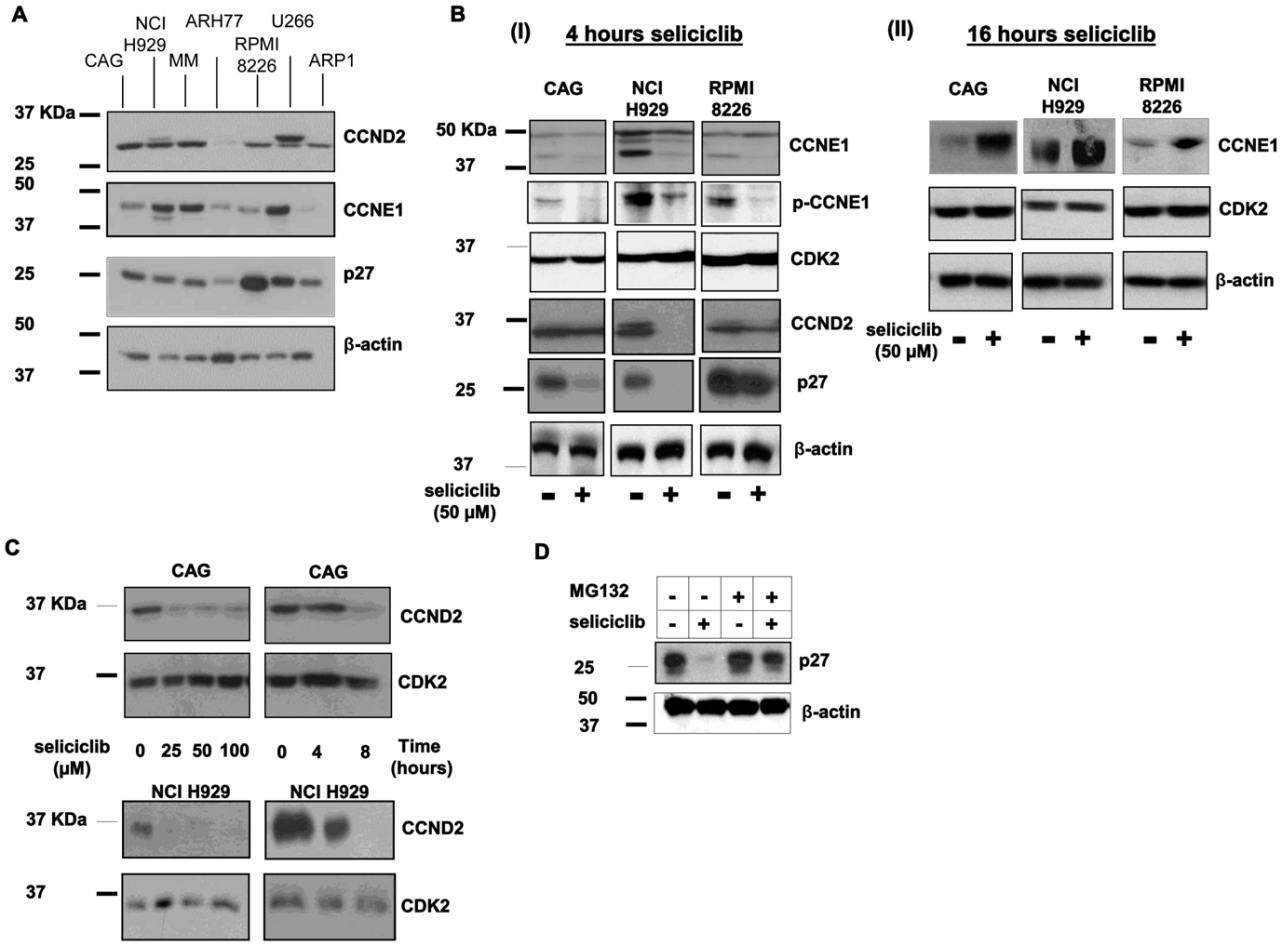
Effect of seliciclib on cell cycle regulators expression in hMMCLs. (A) The indicated multiple myeloma cell lines in logarithmic growth phase were extracted and subjected to immunoblotting, utilizing CCND2, CCNE1 and p27 antibodies. β-actin expression serves as an internal loading control. (B) Seliciclib effect on CCNE1, phosphor-CCNE1, CCND2, CDK2 and p27 expression: the indicated hMMCLs were incubated in the presence or absence of 50 µM seliciclib for 4 (I) or 16 (II) hours. Control cells were incubated in the presence of DMSO. Cells were lyzed and extracts were subjected to immunoblotting, utilizing specific antibodies. (C) CAG and NCI H929 cells were incubated in the absence or presence of increasing concentration of seliciclib for over-night incubation or in 50 µM selicicilb for the indicated time points. Cells were lyzed and extracts were subjected to immunoblotting, utilizing CCND2 and CDK2 antibodies. Control cells were incubated in the presence of DMSO. Experiments were performed 3 times and one representative result is presented**.** (D) CAG cells were incubated in the absence or presence of seliciclib or MG132 (10 µM) exclusively or combined. p27 level of expression was verified by immunoblotting. β-actin was used to confirm equal protein loading.

We were interested in examining the effect seliciclib has on the expression of both cyclins: CCND and CCNE1 as well as p27kip1, a negative regulator of CDK’s. Initial analysis revealed a heterogeneous expression profile of the various hMMCLs (Figure 5A). CCND2 was detected mainly as a 33 kDa protein with additional higher molecular weight bands. We defined the hMMCLs according to the level of CCND2 expression: high (CAG, MM, NCI-H929, and U266), medium (RPMI8226 and ARP1) and low (ARH77).

Comparison of the levels of CCND2 and CCNE1 expression revealed variability between the different cell lines. Whereas all the high CCNE1 expressing lines expressed high level of CCND2, the RPMI8226 low-CCNE1 expressing cell line expressed high levels of CCND2.

The same heterogeneity was observed on the p27 protein expression, some of the cell lines expressed high levels of the protein (RPMI8226 and U266), while others expressed intermediate-low levels (Figure 5A). Interestingly, an inverse relation was detected between the level of CCNE1 and its inhibitor p27 in some of the hMMCLs; high-CCNE1 expressing NCI-H929 had low levels of p27 and low-CCNE1 RPMI8226 had high levels of p27.

Next, the effect of seliciclib on these key cell cycle regulators was examined. Cells were incubated in the presence or absence of seliciclib (50 µM) for 4 hours and the level of protein expression was determined by immunoblotting. For comparison the levels of CCND1 as well as CDK2 remained unchanged within each cell line. Dramatic reduction in the level of CCND2 and p27 was detected in all cell lines (basal levels of ARH77 were too low to detect a decrease following inhibitor-treatment) (Figure 5B I). The seliciclib-induced decrease in the level of CCND2 was dose and time dependent (Figure 5C).

The expression profile of CCNE1 (Figure 1A) revealed several migrating bands reflecting post-translational modifications. Phosphorylation of CCNE1 on Ser-399 by CDK2 was reported to accelerate degradation via the ubiquitin proteasome pathway [32]. Short exposure to seliciclib did not affect the levels of the full-length p50 form of CCNE1. However, the faster migrating bands of CCNE1 disappeared in seliciclib-treated cells suggesting that CCNE1 was not phosphorylated in the presence of CDK inhibitor, whereas the level of non-phosphorylated CCNE1 remained unchanged following 4 hours of seliciclib treatment (Fig. 5B I). It is conceivable that the seliciclib-induced decrease in p27 levels may lead to subsequent stabilization of CCNE1. To test this hypothesis, we evaluated CCNE1 levels following prolonged (16 h) exposure to seliciclib. Indeed, significant increase in the level of CCNE1 was detected after a long incubation period with seliciclib (Figure 5 B II).

To further elucidate the possible effect of seliciclib on CCNE1 phosphorylation we evaluated the levels of phosphorylated CCNE1 in seliciclib-treated hMMCLs. We observed a decrease in CCNE1 phosphorylation following short (4 h) seliciclib treatment, whereas total levels of CCNE1 were not significantly affected by seliciclib (Figure 5C).

To explore the seliciclib-induced down regulation of p27 we incubated the cells in the presence of both seliciclib and MG132. In the presence of both inhibitors, the seliciclib-induced decrease in p27 expression was eliminated, with a pattern similar to MCL1 expression response (Figure 4E). These results suggest that the seliciclib-induced apoptosis is accompanied by down-regulation of p27, as well as MCL-1 protein via the proteasome-mediated degradation pathway, decrease of phosphorylated CCNE1 level and stabilization of total CCNE1.

### Combination of Seliciclib with Flavopiridol Elicits Enhanced Anti-myeloma Activity

**Figure 6:**
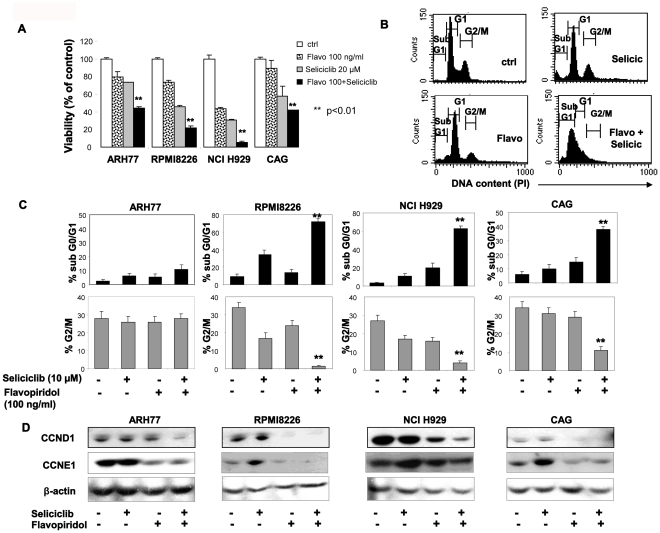
Effect of seliciclib anf flavopiridol combined treatment on hMMCLs viability, cell cycle distribution and cyclin expression. (A) The indicated myeloma cell lines were incubated in the absence or presence of 20 µM seliciclib, 100 ng/ml flavopiridol or combination of both agents for 2 days. Cell viability was determined by MTT assay. Data are represented as mean±standard deviation. Probability values of t-test are presented **p<0.01. (B–C) Cell cycle analysis by PI staining was performed on ARH77, RPMI8226, NCI H929 and CAG cells cultured in the absence or presence of 10 µM seliciclib, 100 ng/ml flavopiridol or combination of both agents for 2 days. (B) Representative cell cycle distribution in NCI H929 cells, treated with seliciclib, flavopiridol or their combination. (C) Combined treatment with seliciclib and flavopiridol increases subG1 and decreases G2/M fractions in hMMCLs. Data are represented as mean±standard deviation. Probability values of t-test are presented **p<0.01. (D) Effect of seliciclib and flavopiridol on CCND1 and CCNE1 expression. Cells were incubated in the presence of 25 µM seliciclib, 100 ng/ml flavopiridol or combination of both for 8 hours. Cells were lyzed and extracts were subjected to immunoblotting, utilizing CCND1 and CCNE1 antibodies. β-actin was used to confirm equal protein loading.

To further explore the role of CDK in hMMCLs survival and the expression of cell cycle regulators we combined seliciclib treatment with flavopiridol in order to achieve a more prominent CDK inhibition. Flavopiridol is a semisynthetic flavonoid that potently inhibits various CDKs, including CDK7 and CDK9. It effectively promotes tumor cell apoptosis [33] and was the first CDK inhibitor to enter clinical trials [34]. We combined low doses of flavopiridol (100 ng/ml) with low doses of seliciclib (10–20 µM) and evaluated the effect of combined treatment on hMMCLs viability. As shown in figure 6A, combination of the two CDK inhibitors significantly reduced the number of viable cells in culture, in comparison to each inhibitor alone. Furthermore, combination of seliciclib with flavopiridol resulted in increased cell cycle alterations and apoptosis in hMMCLs (Figure 6B). Combination of both agents effectively increased the subG1 (apoptotic) population, accompanied by subsequent decrease in the percentage of G2/M cells (Figure 6C). Maximal combined effect on apoptosis induction was achieved in seliciclib-sensitive NCI H929 and CAG cells. In NCI H929 cells combined treatment resulted in 63% of subG1 cells, vs.3.2% in control cells, 11% induced by seliciclib (10 µM) alone and 20% induced by flavopiridol (100 ng/ml) alone. In CAG cells the combined treatment yielded similar results, showing 62% of subG1 cells, whereas seliciclib and flavopiridol alone resulted in 10% and 17% subG1 cells, respectively (Figure 6C). In contrast, seliciclib-resistant ARH77 cells did not respond to low concentrations of seliciclib and flavopiridol, demonstrating only a mild increase in subG1 population following combined treatment (2.7% in control, 6% in seliciclib- and 5.5% in flavopiridol-treated cells vs. 11% in the combined protocol). Notably, elevated dose of flavopiridol (500 ng/ml) increased the apoptosis of ARH77 cells, yielding 21% of subG1 population when applied alone and 35% of subG1 cells in combination with 10 µM of seliciclib (data not show). Moreover, by combining flavopiridol with seliciclib we were able to show effective decrease in CCND1 and CCNE1 expression, evaluated by immunoblotting (Figure 6D). The most prominent effect of the combined treatment on CCND1 expression was detected in ARH77 and NCI H929 cells. Importantly, flavopiridol treatment efficiently decreased the basal and seliciclib-induced levels of CCNE1 in MM cells. These results may explain the mechanism of the observed increased toxicity induced by the combination of seliciclib with flavopiridol. All together, these data indicate that combined CDK inhibition effectively promotes apoptosis and reduces expression of cell cycle regulators in hMMCLs.

### Over-expression and Silencing of CCNE1

**Figure 7:**
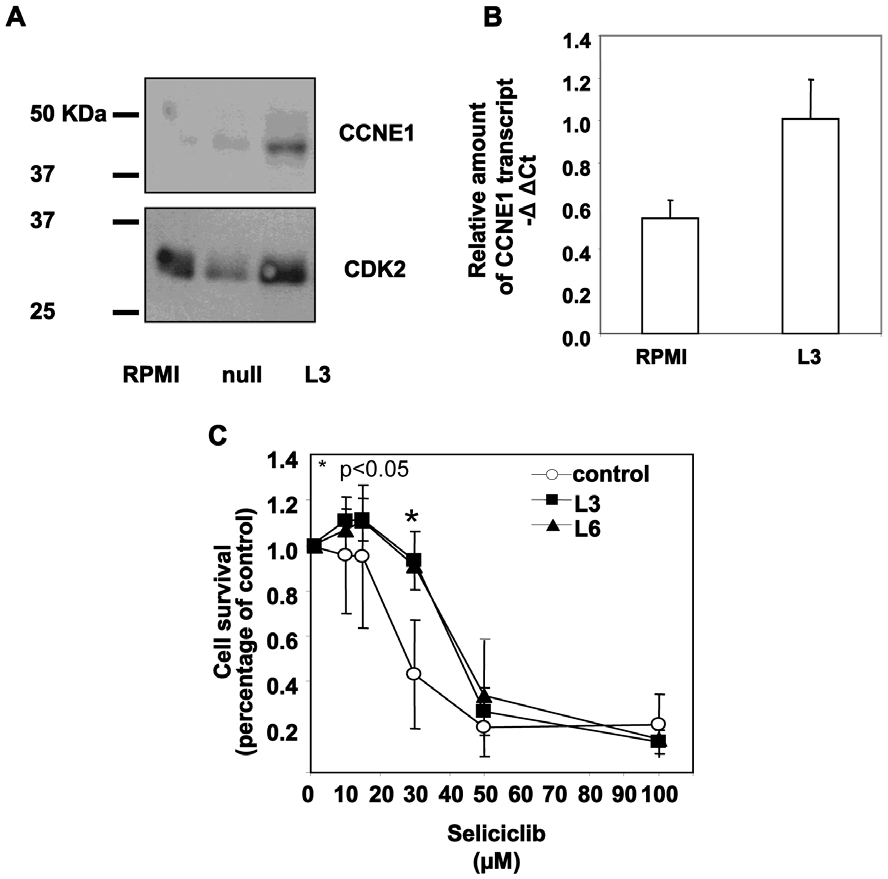
CCNE1 over-expression. RPMI8226 a low-CCNE1-expressing cell line was stably transfected with pRCCMV2hCyclin E1. (A) Stable clones have been selected by addition of G418, and cell extracts of selected cloned were subjected to immunoblotting, utilizing CCNE1 antibodies. CDK2 expression serves as an internal loading control. (B) Stable clones have been selected by addition of G418, and cell extracts of selected cloned were subjected to qRT-PCR. Presented is the relative amount of CCNE1 as compared to the levels of GAPDH**.** Data are represented as mean±standard deviation**.** (C) CCNE1-overexpressing stable cell lines as well as paternal cell line were incubated in the absence or presence of increasing concentrations of seliciclib for 3 days. Cell viability was determined by MTT assay. Data are represented as mean±standard deviation. Experiments were performed twice and one representative result is presented.

In light of the heterogeneity in CCNE1 level of expression in the various hMMCLs, we were interested in exploring the effect over-expression of CCNE1 may have on the sensitivity to CDK inhibitors. To this end, we chose a low CCNE1-expressing hMMCL RPMI8226 and stably transfected it with a CCNE1 expressing plasmid, stable clones have been selected by addition of G418. The selected clones expressed an increased level of CCNE1 in comparison to the cells transfected with vector only (Figure 7A). The level of expression was moderate and was comparable to NCI H929 cells expressing constitutive high endogenous level of the protein. Similar increase in CCNE1 level of transcript was detected by qRT-PCR in the transfected cells (Figure 7B).

We then addressed the effects over-expression of CCNE1 may have on sensitivity to seliciclib. Several clones stably expressing CCNE1 were incubated in the absence or presence of increasing concentrations of seliciclib for 72 hours and assayed for cell viability by MTT assay. Figure 7C depicts that the cells exhibited a decreased sensitivity to the drug as compared to the paternal cell line RPMI8226.

**Figure 8:**
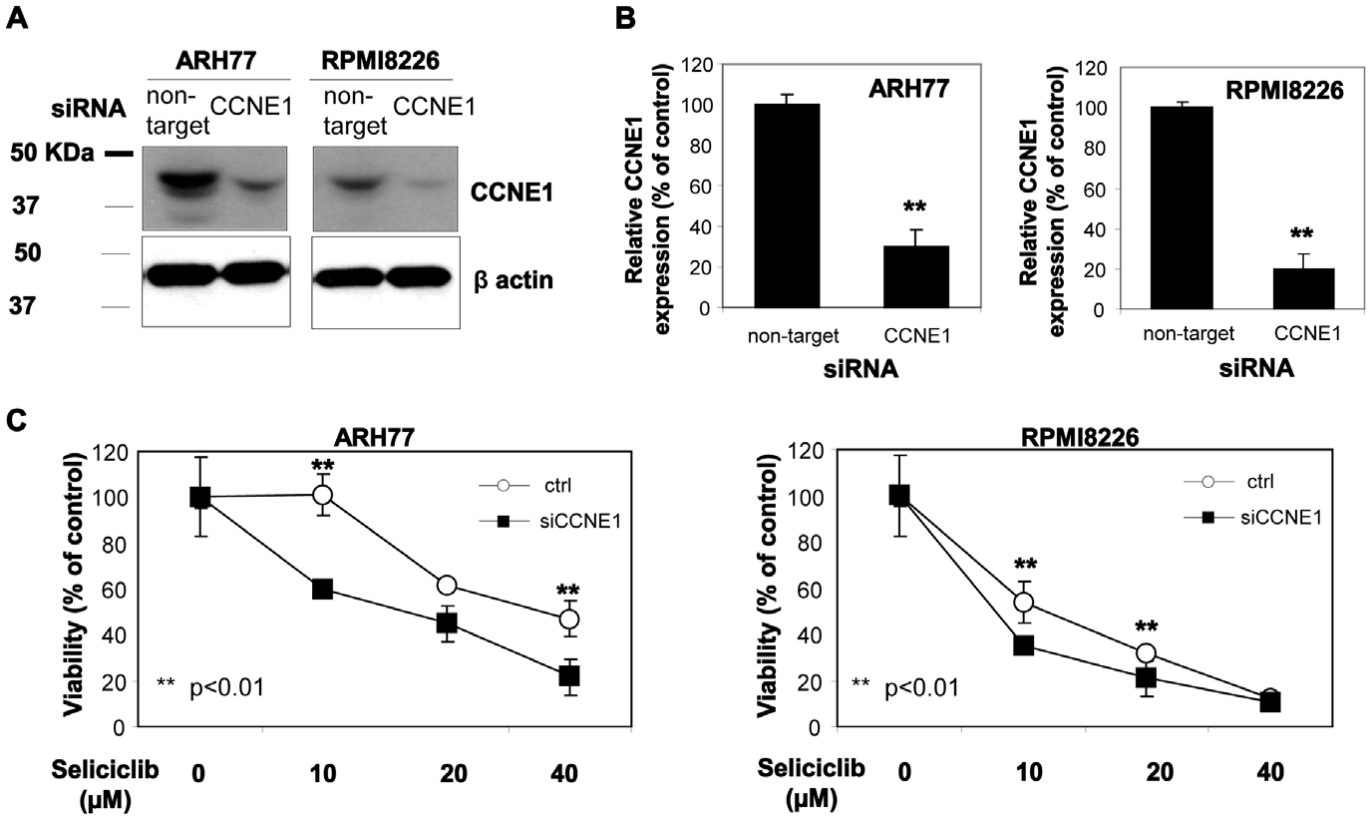
CCNE1 silencing. ARH77 and RPMI8226 cells were transfected with control or CCNE1 siRNA (500 nM) using electroporation. (A) 48 hours following the transfection the cells were lyzed and subjected to immunoblotting, utilizing CCNE1 antibody. β-actin was used to confirm equal protein loading. (B) Quantification of CCNE1 protein levels 48 hours post transfection using TINA software. Data represents the mean and standard deviation of two different experiments. Probability values of t-test are presented **p<0.01. (C) At 48 hours following the transfection the cells were treated with seliciclib (10–40 µM) for additional 48 hours. Viability was determined using MTT assay. Data are represented as mean±standard deviation (**p<0.01). Experiments were performed twice and one representative result is presented.

To further investigate the role of CCNE1 levels in sensitivity to CDK inhibition with seliciclib, we silenced CCNE1 expression in high CCNE1-expressing seliciclib-resistant ARH77 and low CCNE1-expressing seliciclib-sensitive RPMI8226 cells using CCNE1 siRNA. Cells were electroporated with control non-target siRNA or specific anti-CCNE1 siRNA and significant reduction (more than 70% comparing to control) in CCNE1 protein levels was observed following 48 hours in both cell lines, verified by immunoblotting (Figure 8A, B). Next, we exposed control and CCNE1-silenced cells to elevated concentrations of seliciclib for 48 hours and evaluated the viability using MTT. Cells with silenced CCNE1 demonstrated higher sensitivity to seliciclib comparing to control cells (Figure 8C).

These results suggest that expression levels of CCNE1 are able to alter the sensitivity of hMMCLs to seliliclib-induced apoptosis.

### Cell Adhesion and CDK Activity

**Figure 9:**
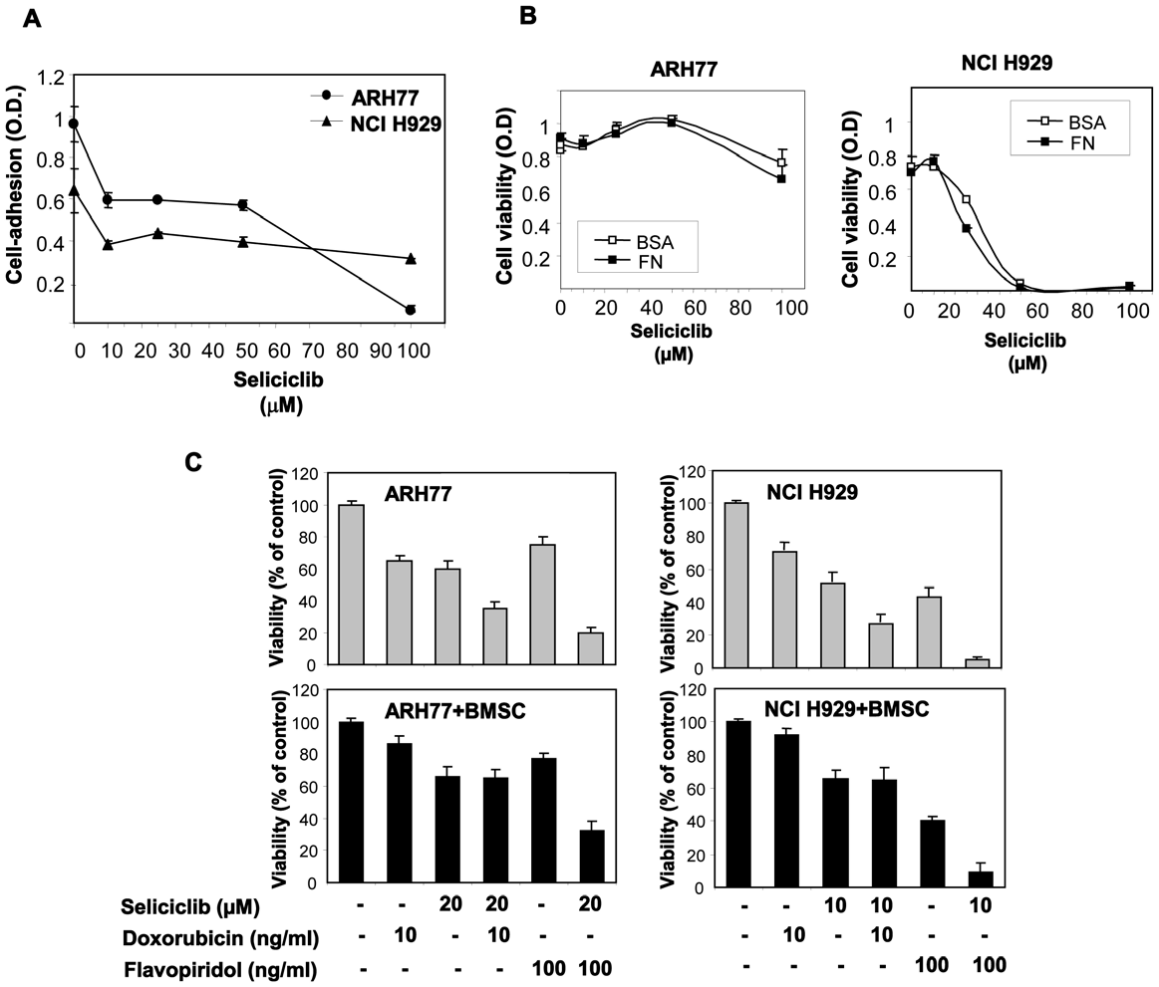
Seliciclib effect on adhesion and cytotoxicty. (A) The indicated cells were pre-incubated in the absence or presence of increasing concentrations of seliciclib for one hour, following which the cells were plated onto 40 µg/ml fibronectin coated plates. Following one hour incubation, suspended cells were removed by repeated washes. Quantification of the adherent cells was performed by crystal violet staining. Data are represented as mean±standard deviation. (B) The indicated cell lines were allowed to adhere to 40 µg/ml FN-coated plates for one hour, following which they were exposed to increasing concentrations of seliciclib for three days. Cell viability was determined by MTT assay. Data are represented as mean±standard deviation. (A–B) Experiments were performed at least 3 times and one representative result is presented. (C) ARH77 and NCI H929 were incubated in the absence or presence of BMSCs, treated with seliciclib (10 µM for NCI H929 and 20 µM for ARH77), doxorubicin (10 ng/ml), flavopiridol (100 ng/ml) or combinations during 48 hours. Cell viability was determined by MTT assay. Signal produced by BMSCs alone was subtracted as background.

Cytotoxic activity against multiple myeloma is known to be hampered by various mechanisms resulting in drug resistance. Adhesion to extracellular matrices, including fibronectin (FN) has been associated with cell-adhesion-mediated drug-resistance (CAM-DR) [35], [36]. As MM cells are embedded in the bone-marrow and rely on stromal microenvironment for their survival, such mechanisms of CAM-DR are of high significance in treating MM. We therefore investigated the effect of seliciclib on cell adhesion to FN. Cells were pre-incubated for one hour, in the absence or presence of increasing concentrations of seliciclib, following which they were subjected to cell adhesion assay. Seliciclib reduced the adhesion of high adhesive cells ARH77 as well as medium adhesive cells NCI H929 (Figure 9A). Similar results were obtained with all the hMMCLs (data not shown). These results show that seliciclib has a direct inhibitory effect on adhesion and displays an additional advantage as a chemotherapeutic drug.

Since cell adhesion is known to render cells insensitive to cytotoxic drugs by mechanisms of CAM-DR [36] we wanted to explore the cytotoxic effect of seliciclib on adherent cells. Cells were seeded onto FN or BSA coated 96 well plates and cell viability was assayed 72 hours after the addition of increasing concentration of seliciclib. Whereas there was a protective effect of FN-adhesion against doxorubicin, a known drug that is displays CAM-DR (data not shown), there was no significant difference between the cytotoxic effect of selicicilib on FN-adherent cells and suspended cells (Figure 9B).

In addition, we evaluated the anti-myeloma effect of seliciclib in combination with doxorubicin. Cells were treated with low concentrations of seliciclib (20 µM for ARH77 and RPMI8226 cells and 10 µM for NCI H929 and CAG cells) and 10 ng/ml of doxorubicin during 48 hours, viability was tested by MTT. Significant increase in cell cytotoxicity was observed following combined seliciclib-doxorubicin treatment (Figure 9C).

We next examined the effect of doxorubicin and flavopiridol, each agent alone or in combination with seliciclib, on hMMCLs co-cultured with bone marrow stromal cells (BMSCs). As shown in Figure 9D, BMSCs protected myeloma ARH77 and NCI H929 cells from doxorubicin-induced cytotoxicity. Seliciclib effectively targeted myeloma cells in the presence of BMSCs. However, in co-culture with BMSCs same doses of seliciclib were not potent enough to overcome the stroma-induced doxorubicin resistance in both ARH77 and NCI H929 cells. In contrast to doxorubicin treatment, combination of seliciclib with flavopiridol efficiently reduced the viability of myeloma cells in co-culture conditions, successfully overcoming the protective effect of stroma (Figure 9D).

Taken together these results indicated that MM are sensitive to seliciclib regardless of their adhesive property. Moreover, adhesive cells are not rendered resistant to seliciclib and do not display CAM-DR, due to the direct prevention of cell adhesion by the drug. Combination of seliciclib with another CDK inhibitor flavopiridol may effectively overcome stroma-mediated resistance and therefore serve as possible anti-myeloma therapeutic strategy.

## Discussion

Proliferation of any given cell is tightly regulated by progression through checkpoints located at the boundaries of cell cycle stages. Progression through each phase of the cell cycle is controlled by the activity of different cyclin-dependent kinases (CDKs) which are heterodimers of a regulatory kinase coupled with a cyclin regulatory subunit [37]. The Cyclin level of expression oscillates throughout the cell cycle, enabling the timely coupling of the distinct dimers. The association of CDK4 or CDK6 with D-type cyclins is critical for the G1 phase progression, whereas the CDK2-cyclin E complex is important for initiation of the S phase [38]. Deviation from the orderly control can and does lead to a neoplasm [19]. The most common translocations in multiple myeloma, lead to dysregulation of CCND, an over-expression with yet unknown biological significance [18]. The profile of CCNE1 in MM and its involvement in this disease are the focus of the current study. We present data displaying heterogeneity of CCNE1 expression in various MM cell lines. We show that ectopic over-expression of this protein promotes the MM cell resistance to seliciclib, a CDK inhibitor with high affinity towards CDK2/CCNE, whereas CCNE1 silencing with siRNA increases the cell sensitivity to seliciclib. This inhibitor is currently under phase II clinical trials. We also discovered an additional potential benefit of seliciclib, namely that it inhibits cell adhesion.

The expression of cyclin E is controlled by both transcriptional and post-translational levels of regulation. At the transcriptional level CCNE1 is directly regulated by E2F, which is sequentially regulated by CCND and CCNE1 through the removal of the inhibitory regulation of pRb [39], [40]. At the post-transcriptional level CCNE1 is regulated by several means: binding to CDK, autophosphorylation and degradation. The CCNE stability is regulated by its binding to CDK which protect the protein from degradation. Only the monomeric cyclin is degraded. Upon binding to CDK, CCNE undergoes phosphorylation on 2 specific degron motifs [32] which results in binding of an additional ubiquitin ligase Fbw7 unit [41]. Ubiquitination and proteosomal degradation follow and lead to downregulation of CCNE and CDK activity.

It is evident that this meticulous regulation of the stability of CCNE is critical for a normal cell cycle progression. Indeed, accumulation of CCNE either by over-expression or by defective degradation is observed in several types of cancer amongst which are breast, lung, cervix, endometrium, gastrointestinal tract, sarcomas and hematological malignancies such as lymphoma and leukemia (reviewed by Hwang et al.) [38]. Moreover, CCNE1 expression was found to be a prognostic marker related to the clinical outcome. High expression, as detected by western blotting, correlates with worse patient outcome for breast, small cell lung carcinoma, squamous cell carcinoma of the larynx and adenocortical tumors. Notwithstanding the prognostic value of CCNE1, its significance as a single event in the progression of a neoplasm needs to be established. We found high expression of CCNE1 in several multiple myeloma cell lines at the level of the transcription. Interestingly, we did not find a direct correlation between the levels of CCND and CCNE1, namely cells such as NCI H929 express high level of both proteins whereas ARP1 and CAG that express that same level of CCND express low levels of CCNE1. As CCND expression is unregulated in most MM patients [42], E2F is positively regulated and hence the CCNE1 levels of expression are expected to increase. This discrepancy could be explained if various regulatory mechanisms of transcription inhibition and/or enhanced degradation pertain. The elucidation of these mechanisms should be addressed of future studies.

Oscillation of the regulatory cyclin is one level of CDK regulation, further regulation is brought about by a family of CDK-inhibitors; these proteins bind to and inhibit the kinase activity of the CDK. Altered expression of p27kip, a member of the CDK inhibitor family, has been shown to accompany cancers, via various mechanisms of impaired synthesis, accelerated degradation and mislocalization [43]. p27kip1 has been reported to have low expression in MM patients as opposed to its high expression in normal plasma cells, reflecting the importance of a balanced CDK activity in the proper cell cycle [37], [44].

We show an inverse relation between the expression levels of CCNE1 and p27, which correlates to the interdependence of both proteins [45]. Whereas, binding of p27 inhibits CDK activity but promotes CCNE1 stability; phosphorylation of p27 by CDK2/CCNE1 stimulates its ubiquitination by the SCF-Skp2, leading to its proteasomal degradation [46], [47].

### Pharmaceutical Inhibition of CDK

CDKs have a central role in cell cycle progression and have been for long a desired target for chemotherapeutic inhibition [19]. This notion was fortified when tumor cells were found to be more sensitive to the inhibitors than normal cells allowing for selective destruction of neoplasmic cells.

The lack of toxicity in normal cells may be explained by the unforeseen findings that CDK2, CDK4 and CDK6 are dispensable for normal mouse development. Knock-out mice of each CDK are viable and display normal cell cycle progression, revealing the dispensability and redundancy of these kinases. Therefore in normal conditions, inhibition of a specific CDK may be compensated by the activity of other CDKs.

Multiple myeloma has been found to be sensitive to CDK inhibition by various chemical inhibitors including flavopiridol [21], [22], SNS-032 [24], [48], P276-00 [25] and seliciclib [26]. Incubation of hMMCLs with these chemical induce cell cycle arrest and apoptosis. The mechanism underlying the apoptotic death of MM cells was found to be the downregulation of an anti-apoptotic protein MCL1 [21], [22], [25], [26]. Our results confirm the above and show downregulation of MCL1 that precedes the cell death induced by CDK inhibition. Transcription of MCL1 was shown to be reduced by CDK inhibition due to suppression of RNA polymerase II [21], [22], [24]–[26].

We provide evidence that in addition to the previously reported inhibition of transcription, post-translational modification of MCL1 renders its stability and targets it for protein degradation. Upon selcliclib treatment, MCL1 separation on SDS-PAGE indicates post-translational modification. A direct correlation was found between the stability of MCL1 and its phosphorylation. Whereby, MCL1 undergoes distinct phosphorylation events at the PEST region (Thr 163), which protects it from rapid degradation [31]. In this study, treatment with seliciclib yielded a dephosphorylated form of MCL1 which was easily degradable and was therefore present at reduced levels. Furthermore, we observed significant decrease in phosphorylated MCL1 levels following seliciclib treatment, detected by specific anti-phospho-MCL1 (Ser159/Thr163) antibody. We show that inhibition of proteasome degradation indeed results in accumulation of MCL1. It is interesting to note that in spite of stabilized MCL1, the inhibition of proteasomal degradation by seliciclib induces apoptosis in MM cells [49].

Plasma cell leukemia cell line ARH77 had undetectable levels of MCL1 even prior to CDK-inhibition, suggesting an additional mechanism of cell death induction alongside MCL1 downregulation. An additional antiapoptotic protein, XIAP was found to be sensitive to CDK inhibition in Chronic Lymphocytic Leukemia (CLL) [50]. It would be interesting to verify whether XIAP is expressed in ARH77 and is regulated by seliciclib treatment.

In addition to the downregulation of MCL1, we report on down regulation of p27 by CDK inhibition. It is of interest that in p27–/– fibroblasts, the rate of apoptosis is markedly increased [51]. Restoration of p27 expression rescued the cells from apoptosis. Therefore, the reduction in p27 amount, reported herein can lead to the increased seliciclib-induced apoptosis.

An opposite effect of seliciclib on p27 was reported in a xenograft model of breast cancer. Seliciclib arrested the cells at the G2-M phase concomitant with an increase in the expression of p27 [52]. The explanation for this discrepancy possibly lies in the stage of cell cycle arrest and the corresponding active CDK. Where the active heterodimers in G2-M phase are cyclin A/B CDK 1 and in S-phase cyclin E CDK2, all of which are sensitive for seliciclib.

### CCND Over-expression and CCNE

The effect of CCNE1 heterogeneity on seliciclib-sensitivity revealed that over-expression of CCNE1 in a low-expressing cell line resulted in increased resistance to the inhibitor. This possibly can be explained by the higher level of active heterodimres, hence the need for more inhibitor. Interestingly, ectopic expression of CCND1 in hMMCL U266 resulted in higher sensitivity to CDK inhibition albeit a slightly lower expression of the protein [53]. The ectopic expression was accompanied with a change in the cell cycle distribution of the cell population - an increase in S-phase fraction that was insensitive to CDK inhibition, possibly through the prevention of E2F inactivation. This enhanced entry into S-phase under CDK inhibition mediates the increased lethality. The difference between these results and ours could reflect the higher affinity of seliciclib to CDK2/CCNE1 than to CDK4/CCND1, as well as the different cell lines tested, the CCND1 over-expression was tested in U266 and whereas the CCNE1 in RPMI8226. These cells express CCNE1 in an opposite manner whereby U266 expresses high level and RPMI8226 low levels of the protein.

A note of precaution, as there was no correlation in hMMCLs between the level of endogenous expression of CCNE1 and the sensitivity to seliciclib, its levels cannot predict the sensitivity of MM cells to the drug. The complexity of cell cycle regulation in the intact cell, namely heterogenic expression of CCNE1 is not the only parameter that differs between the different patients. Similarly, a recent study utilizing a novel CDK inhibitor P276-00, over-expression of CCND1 resulted in increased resistance to the drug, though there was no correlation to levels of endogenous CCND expression in numerous MMCLs [25].

We and others present data that CDK inhibition downregulates CCND2, interestingly, this effect is not collective to all hMMCLs. CCND2 was downregulated by flavopiridol in ARP1 and ANBL-6 cell lines but not in RPMI8226 [21]. In an additional study CCND1 was the isoform that was sensitive to CDK inhibition, seliciclib resulted in a decrease in its expression in colon cancer cells [28]. It may be different in the case of MM cells, as CCND1 is over-expressed due to t(11;14) (q13;q32) translocation in MM plasma cells and therefore may be free of CDK regulation. However, we revealed that CCND1 and CCNE1 were reduced in the presence of flavopiridol, and in some cell lines addition of seliciclib in combination with flavopiridol further decreased CCND1 and CCNE1 levels.

In contrast to the seliciclib-mediated down regulation of MCL-1 and CCND2, we report of marked increase in CCNE1 expression following prolonged seliciclib treatment. The direct reason for the accumulation of CCNE1 may be the elimination of the negative auto regulatory loop, whereby CDK2 phosphorylates CCNE and targets it for ubiquitination and degradation [32]. There may be an additional correlation between the increased CCNE1 expression and seliciclib-induced apoptosis, a striking increase in apoptosis was also reported in transgenic mice that overexpress CCNE due to elimination of 2 degron signals of CCNE and its accumulation [54]. Consistent with these data, substantial induction of CCNE1 protein and CCNE1/CDK2 kinase activity in multiple myeloma and lymphoma cells following radiation was demonstrated [55]. This increase of CCNE levels might be implicated in the initiation phase of apoptosis, therefore proposing a dual role of CCNE1 in apoptosis of hematopoietic cells [13].

### Adhesion

Normal plasma cells as well as malignant ones reside mainly in the bone marrow [4], this strict location is maintained by cellular crosstalk between the PC and BM cells, as well as factors within the BM such as cytokines, chemokines and growth-factors.

The chemokine receptor CXCR4 and its chemokine SDF-1 ligand mediate homing to the BM [4]. BM setting is also regulated through the interaction of adhesion receptors and their cognate ligands on BM cells and extracellular matrix, the most prominent component of which is fibronectin (FN). We as well as others show that this adhesion is dependent on VLA-4 and that it correlates with its level of expression [35], [56], [57]. Adhesion of tumor cells is known to confer cell-adhesion-mediated-drug-resistance (CAM-DR) that pose therapeutic obstacles in several neoplasms including MM [36], [58]. Therefore, interfering with cell adhesion has been a desired target for increasing cytotoxicty of various chemotherapeutic drugs. Recently, adhesion of MM cells was linked to enhanced STAT-3 mediated IL-6 signaling, supporting further the survival and proliferation of the tumor [59]. We present data adding an advantage to the known effect of seliciclib as an anti MM treatment. Seliciclib cytotoxic effects were comparable whether cells were incubated on FN or BSA, namely there was no CAM-DR. Furthermore, we show that cell adhesion was prevented by increasing dose of seliciclib, possibly explaining the lack of CAM-DR. The mechanism of the adhesion inhibition will be addressed in our future studies. This direct effect of seliciclib on adhesion suggests that seliciclib may be used as a single agent or in combination with other potent drugs that display CAM-DR.

Moreover, we demonstrated that combination of seliciclib with flavopiridol was highly effective in overcoming stromal-promoted drug resistance.

We conclude that Multiple Myeloma exhibits increased sensitivity to CDK inhibition that may proceed via several mechanisms:

1. CCND over-expression is a postulated early pathogenetic event in MM, the survival of this neoplasm depends on its expression. Indeed, seliciclib-induced down regulation of CCND has been shown by us as well as by others.
2. The seliciclib effect was not limited to CCND, it also dramatically reduced the expression of p27. Within a normal cell cycle, the reduction of both proteins accompanies the G1 to S transition. An additional response to seliciclib is an increased expression of CCNE1, which is another marker for S-phase entry. The entry to the S-phase may render the cells to higher susceptibility to the cytotoxic effects of seliciclib.
3. The elevated expression of MCL1 is a poor prognostic marker for Multiple Myeloma. Our results indicate that CDK inhibition represses the expression of MCL1 both on the level of transcription and protein stability. Elimination of this crucial anti-apoptotic protein by anti-sense, has been shown to lead to apoptosis [26], [60], [61].
4. CDK combined inhibition effectively targets MM and overcomes microenvironment-produced resistance, therefore potentially providing the rationale for CDK inhibition as part of therapeutic strategy in myeloma.

## Materials and Methods

### Cells

Human multiple myeloma cell lines (hMMCLs) were obtained from American Type Culture Collection (Rockille, MD, USA): U266, ARH77, RPMI8226, NCI H929. CAG and were a kind gift of Prof. Vlodavsky from Technion. hMMCLs were maintained in RPMI 1640 supplemented with 10% non deactivated FCS, 0.4% glucose, HEPES 10 mM, sodium pyruvate 1 mM and penicillin-streptomycin and incubated at 37°C in a humidified incubator under atmosphere of 5% CO2 and 95% air. NCI-H929 required the addition of beta-meracptoethanol (0.0014%).

The bone marrow stromal cells (BMSCs) were isolated from the BM-mononuclear cells (BM-MNC) by plate adherence. Briefly, BM-MNC were re-suspended in 5 mL of MSC growth medium (MGM) (MEMα supplemented with 20% fetal bovine serum and 1% 100×penicillin–streptomycin), transferred to a 25-cm^2^ flask and incubated overnight. On the next day, after the medium was removed, the adhered cells were washed three times with PBS and grown further in MGM. BMSCs were passaged after reaching 80% confluency by trypsinization.

### Antibodies and Reagents

All antibodies were purchased from Santa-Cruz (Santa Cruz, CA, U.S.A.). Tissue culture reagents were purchased from GIBCO (Carlsbad, CA, USA). Seliciclib was purchased from LKT Laboratories, Inc. (St. Paul, MN, USA) and dissolved in dimethylsulfoxide (DMSO) as a stock solution of 100 mM. Flavopiridol was purchased from Enzo Life Sciences (Farmingdale, NY, USA). All other reagents were supplied by Sigma (Rehovot; Israel).

### SDS-PAGE, Western Blotting

Cells were lyzed in RIPA buffer (25mM Tris pH = 7.6, 150 mM NaCl, 1% NP40, 1% Sodium deoxycholate, 0.1% SDS) plus protease inhibitor cocktail I and II and centrifuged at 10,000 xg for 10 min. Extract supernatant corresponding to 50 µg was loaded per lane. Following SDS-PAGE, the proteins were transferred to nitrocellulose membranes and the membrane was blocked with either 5% skim-milk or 3% BSA in 10 mM Tris, 150 mM NaCl for one hour at rT. Primary antibodies were incubated overnight at 4°C, followed by multiple washes in TBS-T. The appropriate horseradish peroxidase-conjugated secondary antibodies were incubated in the blocking solution for one hour at room temperature (rT), followed by multiple washes with TBS-T. Chemiluminescence was detected using an ECL kit Pierce (Rockford, IL, USA) in accordance with the manufacturer’s protocol.

### Real-Time RT-PCR

Total RNA was extracted from 2×10^6^ cells incubated in the indicated conditions, using nucleospin RNA II Macherey-Nagel (Duren, Germany) according to the manufacturer’s instructions. The RNA was reverse transcribed using the High Capacity cDNA RT Applied Biosystems (Foster City, CA, USA). Quantitative PCR for human CCNE1 (NM_001238.2) was carried-out by utilizing Taqman Assay on demand and the PCR master mix for real-time, Applied Biosystems (Foster City, CA, USA). The PCR products were produced and quantified in 7500 real time PCR system, Applied Biosystems (Foster City, CA, USA).

### Cell Viability Assay

Measurements of cell viability were performed by an MTT assay which quantifies the reduction of MTT with metabolically active cells. Cells were plated in 96-well plate and incubated in the presence of increasing dose of seliciclib or DMSO for 3 days. Cells were pulsed with MTT 5 mg/ml for 4 hours, followed by lysis with 10% SDS, 0.01 M HCl and incubation at 37°C over-night. Absorbance was measured at a wave length of 570 nm using a Molecular Devices spectrophotometer (Sunnyvale, CA, USA).

### Cell Cycle Analysis

Human MMCL cells (0.5×10^6^) were cultured for the specified times in the medium alone, DMSO or with 50 µM seliciclib. The cells were harvested, washed with phosphate-buffered saline (PBS) and fixed with 70% ethanol at –20°C over-night. Fixed cells were treated with 50 µg/mL RNAse for 2 hours at 37°C and then stained with propidium iodide; 50 µg/mL. Cell cycle profile was determined by FACS analysis using BD FACS Calibur multicolor flow cytometer Becton Dickenson (San Jose, CA, USA) and analyzed using Cell quest Becton-Dickenson (San Jose, CA, USA). The subdyploid DNA peak (subG1) representing apoptotic cells.

### Annexin V Binding

The extent of apoptosis was evaluated by flow cytometric analysis utilizing Annexin V staining. Annexin V binding to the exposed phosphatidylserine (PS) at the cell surface occurs early during the process of apoptosis. 1×10^5^ of control or seliciclib-treated cells (50 µM for 4 hours) were stained with the MEBCYTO kit of Annexin V-FITC MBL International (Woburn, MA, USA) according to manufacturer’s protocol. The samples were analyzed using flow cytometry within 1 hr to determine the percentage of cells displaying Annexin V positivity.

### Stable CCNE1-expressing Cell Line

RPMI H929 cells were transiently transfected using TransIT-LT1 Mirus (Madison, WI, USA), with either pRCCMVCycE (a kind gift of Prof. Doron Ginsburg, Bar-Ilan university), or pRCCMV, according to the manufacturer’s suggested protocol. Following the transfection the cells were placed in 1 mg/ml G418. Positive clones were isolated by survival of stable clones in the selection media for over a month. CCNE1-expressing clones were identified by immunoblotting.

### Knock-down of CCNE1 with siRNA

ARH77 and RPMI8226 were transfected with 500 nM of control non-target or specific CCNE1 siRNA (siGENOME SMARTpool, obtained from Dharmacon, Thermo Fisher Scientific, Lafayette, CO, USA) using Amaxa electroporation system (Lonza, Basel, Switzerland) and Ingenio electroporation kit (Mirus) according to the manufacture’s instructions. Forty eight hours following the electroporation the cells were harvested for immunoblotting and CCNE1 level were tested. In parallel, control and siCCNE1-silenced cells were exposed to elevated amounts of seliciclib for additional 48 hours and their viability was then tested using MTT method.

### Adhesion Assay

Fibronectin (FN) or BSA-coated plates were prepared by coating 96-well plates with either (40 µg/ml) of soluble FN or bovine serum albumin (BSA), followed by incubation at 37°C for 2 hours decanting of the liquid and evaporation overnight in a biological hood. Cells were loaded into the coated-plates and incubated for one hour at a humidified incubator at 37°C. Seliciclib-treated cells were initially incubated on BSA-coated plates for one hour and then transferred to FN-coated plates. Unattached cells were removed by washing three times with serum free RPMI media, and fixed with 70% methanol for 10 min at rT. Cell staining was carried out by incubation for 10 minutes at rT with 0.02% crystal violet/0.2% ethanol solution, followed by washes with H_2_O. Solubilization of the stained cells was completed by addition of 2% SDS for 30 minutes at rT. Absorbance was read at a wave length of 550 nm on a spectrophotometer Powerwave300 Bio-Tek Instruments Inc. (Winooski, VT, USA).

Images of cells were captured by an Olympus 2X70 microscope utilizing DP controller software Olympus (Hamburg, Germany).

### Statistics

Statistical analysis was carried out by the student’s t-test. A p-value less than 0.05 was considered to be statistically significant.
